# Cytoreductive prostatectomy: Evidence in support of a new surgical paradigm (Review)

**DOI:** 10.3892/ijo.2014.2656

**Published:** 2014-09-17

**Authors:** IZAK FAIENA, ERIC A. SINGER, CHRIS PUMILL, ISAAC Y. KIM

**Affiliations:** Section of Urologic Oncology, Rutgers Cancer Institute of New Jersey and Rutgers Robert Wood Johnson Medical School, New Brunswick, NJ 08903, USA

**Keywords:** prostate cancer, radical prostatectomy, metastasis, cytoreductive

## Abstract

Prostate cancer (PCa) remains the second ranked cause of cancer deaths in the United States. The current standard of care for metastatic prostate cancer (mPCa) includes systemic therapies with no option for surgery. In contrast, in other malignancies such as breast and kidney cancer, cytoreduction plays an integral role in the treatment of metastatic disease. In this framework, there are emerging data that suggest a potential oncologic benefit to cytoreduction in mPCa. The majority of the data are retrospective in nature suggesting that patients with mPCa who had prior radical prostatectomy (RP) had a better survival, as well as improved response to systemic therapy. Similarly, patients who presented with metastatic disease and received definitive local therapy (RP or radiation) had greater survival than patients who received no treatment. In order to confer maximum potential benefit, operating in the setting of mPCa must be technically feasible with acceptable morbidity. It has been demonstrated in many studies that operating on locally advanced disease (T3a/b) does have similar morbidity as lower stage cancer. This may be applicable in the metastatic setting, because although PCa may have metastasized, it may remain locally advanced. On the molecular level there are a number of explanations concerning the potential benefit of cytoreduction. However, these ideas remain speculative with no concrete evidence to date.

## 1. Introduction

The burden of prostate cancer (PCa) in the United States is estimated to be 233,000 new cases and 29,480 deaths in 2014 alone (1). PCa continues to be the second leading cause of death among men each year (1). While much attention has been given to this common malignancy, it is important to recognize that prostate cancer is not a monolithic entity. Risk stratification allows urologic oncologists to tailor treatment recommendations based on the serum PSA level and biopsy results (2). The high-risk group of PCa patients are the men more likely to benefit from multi-disciplinary interventions (3) as they are more likely to have biochemical recurrence after initial treatment, require secondary treatments, develop metastasis and experience cancer-specific mortality (4).

Patients with metastatic prostate cancer (mPCa) have a poor survival rate of only 30% at 5-years compared to men with non-metastatic PCa, for whom the survival is almost 100% at 5 years (5). Clearly there is a need for additional therapies in the metastatic disease space. While there have been advances in second-line hormonal therapies, immunotherapy and cytotoxic chemotherapy in recent years, androgen deprivation therapy (ADT) remains the foundation of current standard treatment regimens for metastatic disease (6). Although, recent interim analysis from the CHAARTED trial shows benefit of combined treatment with docetaxel and ADT over ADT alone for men with newly diagnosed metastases, adding yet another treatment option in the metastatic setting (NCT00309985). In examining other malignancies with metastasis, there is strong evidence showing improved survival in patients who underwent cytoreductive surgery (7–10). In contrast, the role of cytoreductive prostatectomy has not been rigorously evaluated, largely because of historical concerns (pre-PSA, prior to contemporary imaging techniques), when men often presented with more advanced disease and prostatectomy was aborted when positive lymph nodes were found. However, in recent years, along with advances in surgical technique and staging, there has been some retrospective data suggesting a possible role for cytoreductive surgery in PCa, although the evidence is limited.

## 2. Materials and methods

We performed a non-systematic review of the literature. A PubMed search using the key terms ‘radical prostatectomy’, ‘cytoreductive’, ‘prostate cancer’, ‘metastatic prostate cancer’, ‘high-risk prostate caner’ and surveyed the English language literature for studies looking at this topic. Initial search results yielded 5 studies, which were eliminated based on title and abstract review. Our search was expanded to ‘high-risk prostate cancer’ and ‘radical prostatectomy’, ‘metastatic prostate cancer’, which yielded over 1500 studies. The authors reviewed results, and studies were eliminated by title and abstract review. The authors identified the most relevant studies, which included systematic reviews, studies with the longest follow-up, and studies discussing cytoreduction. We identified further studies by reviewing the reference list of key articles. The search yielded a total of 56 papers, which were reviewed by the authors.

## 3. Indirect evidence in support of cytoreduction

It has been shown that malignancies such as kidney (7), colon (8), breast (9) and ovarian (10) cancers have a clear survival benefit when patients undergo cytoreductive surgery with or without radiation as well as improved response to systemic therapy. However, in mPCa cytoreduction has not been explored. Recent evidence has emerged suggesting a role for cytoreductive prostatectomy in the setting of metastasis. In examining the evidence, some inferences can be made that do suggest a possible benefit. We will survey some of the data.

PCa with positive lymph nodes, considered to be a systemic disease, is usually treated with ADT and/or radiation rather than surgical extirpation (11). Early evidence from Messing *et al*, which was a prospective, randomized study carried out in the pre-PSA era, showed that 14% of patients who underwent RP with pelvic lymph node dissection (PLND) and found to have lymph node-positive (N^+^) disease were disease-free at 12 years without any adjuvant therapy, indicating that some patients with lymph node metastasis can be cured with surgery alone (12). In a population-based study using the Munich Cancer Registry, Engel *et al* (13) examined the OS and relative survival (an estimate of cancer-specific survival) of patients with N^+^ disease undergoing RP (n=688) in comparison with patients in whom RP was aborted (n=250) between 1988 and 2007. They found a superior 10-year OS (64 vs. 28%) and relative survival (86 vs. 40%) in the RP group compared with no RP. In addition, on multivariable analysis, RP was an independent predictor of increased survival [hazard ratio (HR): 2.04; 95% confidence interval (CI), 1.59–2.63; p<0.0001] (13). Earlier studies by Ghavamian *et al* (14) evaluated 79 matched pairs of patients with pT_x_N^+^ PCa undergoing either RP with PLND plus orchiectomy within 3 months of surgery or PLND with orchiectomy only. They found that patients undergoing RP with orchiectomy demonstrated both a higher OS (66 vs. 28%; p<0.001) and a higher disease-specific survival (DSS) (79 vs. 39%; p<0.001) compared with patients undergoing orchiectomy alone.

For locally advanced PCa, studies have shown that reducing the size of the prostate may result in improved overall and progression-free survival. In the largest series, 842 patients with clinical T3 disease were treated with radical prostatectomy 78% of which received adjuvant androgen ablation. In men who received RP, freedom from local or systemic disease at 5, 10 and 15 years was 85, 73 and 67%, respectively, and cancer-specific survival rates were 95, 90 and 79%, respectively (15). Swanson *et al* commented that since recurrence-free survival is related to the incidence of metastatic disease, the above findings support the concept that removing the bulk of the tumor impacts the response to systemic therapy (16). SWOG 8894 was a randomized study that compared orchiectomy alone to orchiectomy plus flutamide (17). Patients enrolled had either been diagnosed with metastatic disease initially or had progressed after surgical castration. Post-hoc analysis revealed that those who underwent prior radical prostatectomy had a better response to androgen ablation and better survival than those with an untreated PCa (HR 0.77; 95% CI, 0.53–0.89). PSA data were insufficient to analyze biochemical recurrence, but patients without a prostate had a better response to androgen ablation. This has become a recurring theme in that patients with less tumor volume respond better to androgen ablation, and most recently, vaccine therapy (17–19).

A recent Surveillance Epidemiology and End Results (SEER) database study analyzed patients with PCa from 1994–2007 in order to elucidate any relationship between primary treatment modality (radical prostatectomy vs. radiation therapy) and cancer-specific survival (CSS) (20). Survival data were stratified for patients who were originally low-risk, and intermediate to high-risk. They found that for the low-risk patients, the adjusted CSS after metastasis was 86.2 vs. 79.3% in the RP vs. RT groups, respectively; for the intermediate to high-risk patients, the CSS after metastasis was 76.3 and 63.3% in the RP vs. RT groups, respectively. The hazard ratios estimating the risk of cancer-specific mortality between the RP and RT groups were 0.64 (95% CI, 0.36–1.16) and 0.55 (95% CI, 0.39–0.77) for the low- and intermediate to high-risk groups, respectively. While the study’s retrospective nature along with other limitations, preclude its generalization to treating the primary tumor after metastasis, it does offer some evidence that controlling the primary tumor may confer survival benefit.

## 4. Direct evidence in support of cytoreduction

Cytoreduction has been shown to be effective in an animal model. Kadmon *et al* (21) implanted R3327/MAT-Lu tumor in a rat model in which lung metastasis developed in all the rats. They treated the rats with either a single dose chemotherapy, surgical excision of the primary tumor, or a combination of tumor excision plus post-operative single dose chemotherapy. Those in which the implanted tumor was removed had increased survival. Of the combined group 42% were long-term survivors (tumor-free >180 days), which was not seen in the non-surgery group. However, they were only able to show in one of the experiments that simply removing the primary tumor resulted in a decrease in lung metastasis. Other studies using the same cell line in a rat model were able to demonstrate that cure was directly dependent on tumor volume (22). It was shown that if a threshold tumor size of >0.5 cc was reached, the rats were not cured by surgery or chemotherapy alone as the majority of rats had already established lung metastases. However, with adjuvant chemotherapy in combination with surgery >90% of the rats were cured as long as the combination approach was initiated when primary tumor was <2 cc in size.

As for human data, a recent SEER database study analyzed the specific question of survival in men with mPCa who received definitive local therapy vs. no therapy (23). They identified 8185 subjects who were eligible for analysis. Median follow-up was 16 months. They found that the 5-year OS and predicted DSS were each significantly higher in patients undergoing RP (67.4 and 75.8%, respectively) or brachytherapy (BT) (52.6 and 61.3%, respectively) compared with patients with no treatment (22.5 and 48.7%, respectively) (p<0.001). Furthermore, undergoing RP or BT was each independently associated with decreased CSM (p<0.01). Moreover, in a separate analysis, they excluded patients who died <12 months after diagnosis in order to reduce the risk of bias of other competing cofounders such as comorbidity, progressive disease or multiple primary malignancies. Again, they found 5-year OS to be higher in patients undergoing RP (76.5%; 95% CI, 67.0–83.7) or BT (58.2%; 95% CI, 44.5–69.7) compared with patients with no treatment (30.6%; 95% CI, 28.9–32.4) (p<0.001) (23). Despite this provocative finding, authors noted a number of important limitations. The study is retrospective in nature with an inherent selection bias towards younger and healthier patients that is expected with surgical therapy. Furthermore, the authors note that some information was unavailable from the SEER database such as patient performance status, comorbidities, site-specific EBRT codes, timing and dosage of chemotherapy and/or ADT, and the use of ADT relative to surgery (23), which could confer benefit to the local therapy group. However, despite these limitations, the results of this study prompt further research in this area.

Most recently, Heidenreich *et al* (24) reported their experience with cytoreductive prostatectomy. They included eighteen patients who were identified to have minimal osseous metastases (equal to or less than three hot spots on bone scan) and the absence of visceral or extensive lymph node metastases. The patients underwent neoadjuvant ADT with luteinizing hormone-releasing hormone (LHRH) agonists for 6 months. Patients then underwent RP only if the PSA serum level decreased to <0.4 ng/ml and their osseous lesions disappeared on repeat bone scan. Following RP, patients received 2 years of ADT. The surgical pathology from these 18 subjects revealed pT2c in 4 (22.2%), pT3a (16.7%) in 3 and pT3b in 11 (61.11%). Seven (38.9%) patients had lymph node metastases and 3 (16.7%) patients had positive surgical margins (PSM). PSM were treated with adjuvant radiation therapy of 66.6 Gy. There were no grade 3–5 complications. The mean follow-up was 29 months (range 3–52). Three (16.7%) patients relapsed, with no evidence of disease in the remaining patients.

Interestingly, Suardi *et al* recently published long-term results on salvage lymph node dissection (LND) in patients with recurrent prostate cancer in lymph nodes as detected by 11C-choline positron emission tomography/computed tomography (PET/CT) scan. (25) They found that approximately 40% of patients remained free of disease at 8 years not requiring ADT, and that patients with lower PSA, pelvic nodal recurrent disease only, and complete biochemical response after salvage LND benefited the most on multivariable analysis. Although these results are interesting, there was no control group in this study, and there were a significant number of patients (60%) with undetectable PSA prior to salvage LND. This concept needs to be explored further.

## 5. Possible mechanisms

To date, the exact mechanism(s) by which cytoreduction in PCa can improve survival have not been elucidated. One important aspect of PCa is that the cells proliferate at a very slow rate with very low rate of turnover, resulting in relatively high rate of survivability for disseminated mPCa cells (26). One potential mechanism of action for cytoreductive prostatecomy is based on the concept of tumor self-seeding. Kim *et al* (27) have elegantly shown that self-seeding of breast cancer, colon cancer and melanoma in mice is preferentially mediated by aggressive circulating tumor cells (CTCs), including those with bone, lung or brain-metastatic tropism. They have also shown that self-seeding can accelerate tumor growth, angiogenesis and stromal recruitment through seed-derived factors. In other words, tumor self-seeding theory suggests that CTCs are the intermediaries between primary tumors and metastases, and that CTCs return to, and grow in, the primary tumor sites from their derived metastases (20). In applying this concept, the prognostic utility of CTC counts in metastatic castrate-resistant PCa was recently evaluated, and results showed that increased baseline CTC count (>5), and rising CTC count portend a worse overall survival (28). This may help guide therapy in this setting, and may be a potential marker for metastatic disease burden. Metastatic cells can also alter expression patterns of the parental tumor site during the metastatic colonization and accelerate tumor progression (29). Perhaps there is a similar phenomenon in PCa. Therefore, it is feasible that removing the primary tumor and reducing overall tumor volume reduces these effects as well as the time to fatal tumor burden which, in turn, may allow other therapies to work more effectively (16). Furthermore, previous studies have shown that the presence and extent of residual tumor cells within the primary site after aggressive treatment may contribute to tumor progression and predict cancer-specific survival (30,31).

Another potential mechanism is the tumor microenvironment which has been suggested to be a source of continued androgen production potentially driving the tumor (32). In addition, suppression of tumoral androgen activity may lead to increased sensitivity to androgens allowing PCa cell survival in a low androgen environment (33). Once again, removing the primary tumor may mitigate these effects leading to improved outcomes.

## 6. Feasibility of the surgery

It is important to demonstrate that risk of operating on patients with advanced disease does not outweigh the potential benefits of local control. To do so, we evaluated the published literature on the role of RP in patients with locally advanced disease (T3a/b disease). This is a reasonable starting point because most men with M^+^ disease distantly will still only have T3a/b disease locally. In the last decade, surgical management of high-risk disease has become more feasible with improvements in preoperative imaging and refinements in surgical technique for both open and robotic radical prostatectomy (34,35). At experienced centers, both surgical approaches offer good functional and oncologic outcomes (Tables I and II) (36,37). Several studies have demonstrated the safety and efficacy of this approach for high-risk PCa.

Berglund *et al* showed that recovery from surgery, duration of catheterization, and the overall return of continence were essentially similar to those observed in the low-risk population (38). Loeb *et al* reported on 288 patients who underwent RP by a single surgeon, of which 52% had pT3 or greater (39). Overall there were 33 (11%) complications, which included cardiac events (2), hematuria (1), inguinal hernias (9), infection (1), anastomotic strictures (11) and thromboembolic events (6). The same group had an earlier study, which also reported similar outcomes (40). Ward *et al* examined 5652 men who underwent RP at the Mayo Clinic of which 842 had cT3 cancer, morbidity was similar to patients with cT2 disease, but direct statistical comparisons were not made (15). Not surprisingly, among patients with very high-risk features (such as PSA >20 ng/ml or hydronephrosis), blood transfusion, operative time and lymphoceles were significantly increased (41). Novara *et al* also reported on 415 high-risk patients who underwent RARP (42). On multivariable analysis, only prostate volume (odds ratio: 0.985; 95% CI, 0.977–0.993; p<0.001) and the number of cases performed (p<0.001) were independent predictors of the occurrence of any grade complications. Agarwal *et al* analyzed 3317 patients in a single institution with a standard reporting of complications found on multivariable analysis, preoperative PSA values and cardiac comorbidity were predictive for medical complications, whereas age, gastroesophageal reflux disease and biopsy Gleason score were predictive of surgical complications (43). However, the authors note that two experienced surgeons did the majority of RARPs. In a population-based study, Gandaglia *et al* evaluated outcomes of high-risk patients undergoing RARP vs. ORP (36). They found comparable outcomes with regard to overall complications (28.3 vs. 30% p=0.6), overall rate of additional cancer therapy (21.2 vs. 24.6% p=0.2) and PSM (21.6 vs. 18.3% p=0.4) with the only significant difference being lower blood transfusion rate (p=<0.001), and shorter length of stay (p=<0.001). In a systematic review conducted by Yuh *et al* looking at the role of RARP in high-risk PCa, also found comparable overall complication rate (3–30%), PSM (35%) with the majority of patients having T3a/b disease (35%, 19%) (44). Other studies (Table I) have also shown similar results.

Salvage radical prostatectomy (SRP) has been demonstrated to be a viable option, technically and oncologically, for patients with radiorecurrent disease (45). Of course, due to radiation effects, RP in the salvage setting carries a higher risk of complications, however with increased experience, and newer techniques these rates are more acceptable (46). In a comprehensive systematic review of salvage radical prostatectomy, Chade *et al* (47) found the most common complications to be anastomotic stricture (7–41%) followed by rectal injury (0–28%) with major complications (Clavien 3–5) ranging from 0–25%. Erectile dysfunction was present in most patients both prior to and after SRP (50–91%, 80–100%), while urinary incontinence rates were between 21 and 90%. Finally, CSS and OS at 10 years were 70–83% and 54–89%, respectively. The conclusion was that in select patients, SRP is a viable option and technically feasible with acceptable morbidity and oncologic outcomes, however, functionally, the outcomes varied significantly. Lessons may be learned from SRP and applied to cytoreduction. In fact, cytoreduction may be less morbid as surgery is being performed on normal tissue.

Currently, a phase II clinical trial (NCT01751438) that is accruing patients at MD Anderson Cancer Center, will randomize patients with hormone-sensitive, M1 prostate cancer to best-systemic therapy or best-systemic therapy plus radiation or surgery with the primary end-point being progression-free survival. Furthermore, patients must have a life expectancy of >2 years, ECOG performance status of 0 or 1, a candidate for surgery or radiation, and finally, initiation of systemic therapy no longer than 6 months prior to randomization. This may help to address the utility of cytoreduction, but the decision of whether to offer radiation or surgery is left to the discretion of the physician, which raises the possible issue of selection bias. Additional prospective studies will certainly be needed.

## 7. Future directions

There are still unanswered questions relating to cytoreductive prostatectomy, namely whether the observations made in retrospective studies will translate to similar findings in a more rigorous clinical trial setting. Furthermore, it is important to explain, at the molecular level, the observations and/or hypotheses as to why local control of PCa may improve overall survival.

Of note, Haffner *et al* tracked the clonal origin of lethal PCa in a 64-year-old man who died from metastatic disease 17 years after radical prostatectomy (48). In this patient, the authors tracked the evolution of his cancer with serial samples until death. Surprisingly, the source of his lethal cancer was from a small, low-grade cancer focus in the original prostatectomy specimen, and not from the bulkier more advanced tumor. This study, although of only one patient, highlights the heterogeneity of PCa, and the need for methodologically sound, large, prospective trials to answer this difficult question.

## 8. Conclusion

PCa remains a challenging disease to treat primarily in the advanced setting. Once metastasized, PCa is treated with systemic therapy with little focus on the primary tumor based on the assumption that the metastases are the driving force of the cancer. However, some intriguing evidence shows a potential benefit in treating the primary tumor as in other malignancies. It has also been shown that locally advanced cancer can be operated on with comparable outcomes lending further support for local control. Taken together, and despite the plethora of weak evidence, there seems to be a foundation on which to build upon in future carefully performed clinical trials regarding the role of cytoreductive prostatectomy in mPCa.

## Figures and Tables

**Table I:** Summary of outcomes of patients undergoing RP for T3a-b PCa.

	Sample size	% Gleason (8–10)	% (+) LN	(+) Margins	% Complication	Mean EBL (ml)	Mean operative time (min)	Hospital stay (days)	Continence (%)	Potency (%)
Connolly *et al* (49)	160	75	15	38		-	-	-	-	-
Gandaglia *et al* (36)	353	48	3	22	28	-	-	2	-	-
Jung *et al* (50)	200	40	9	42	-	250	190	4	-	-
Lavery *et al* (51)	123	81	2.4	31	-	84	147	1.6	78^a^	56^a^
Ou *et al* (52)	148	30	14	53	7.4	100	150	3	95^a^	60^a^
Rogers *et al* (53)	69	62	1.4	42	5.8	150	175	1	82^c^	33^b^
Sagalovich *et al* (54)	82	93	13	12	-	150	111	-	-	-
Yuh *et al* (55)	30	73	33	27	30	200	186	1	-	-
Zugor *et al* (56)	147	100	17	33	14	183	164	-	-	-

EBL, estimated blood loss.

a At 12 months,

b at 26 months,

c at 36 months.

**Table II:** Survival data for patients undergoing RP for high-risk PCa.

	10 Year cancer specific survival (%)
Hsu *et al* (57)	91.60
Freedland *et al* (58)	72–92
Carver *et al* (59)	85
Ward *et al* (15)	73
Gandaglia *et al* (37)	89.90
